# Pig producer perspectives on the use of meat inspection as an animal health and welfare diagnostic tool in the Republic of Ireland and Northern Ireland

**DOI:** 10.1186/s13620-015-0057-y

**Published:** 2016-02-09

**Authors:** Catherine Devitt, Laura Boyle, D. L. Teixeira, N. E. O’Connell, M. Hawe, Alison Hanlon

**Affiliations:** 1 UCD Planning and Environmental Policy, University College Dublin, Belfield, Dublin 4 Ireland; 2 Teagasc Pig Development Department, Animal and Grassland Research and Innovation Centre, Moorepark, Fermoy, Co, Cork, Ireland; 3 Laboratório de Etologia Aplicada e Bem-estar Animal, Universidade Federal de Santa Catarina, Santa Catarina, Brazil; 4 Institute for Global Food Security, Queens University Belfast, Northern Ireland Technology Centre, 18-30 Malone Road, Belfast, BT9 5BN UK; 5 College of Agriculture, Food and Rural Enterprise, Greenmount Campus, Tirgracy Road, Antrim, BT41 4PS UK; 6 School of Veterinary Medicine, University College Dublin, Belfield Dublin 4, Ireland

**Keywords:** Meat inspection, Pig health, Pig producers, Pig welfare, Qualitative research

## Abstract

**Background:**

Currently, there is growing interest in developing ante and post mortem meat inspection (MI) to incorporate measures of pig health and welfare for use as a diagnostic tool on pig farms. However, the success of the development of the MI process requires stakeholder engagement with the process. Knowledge gaps and issues of trust can undermine the effective exchange and utilisation of information across the supply chain. A social science research methodology was employed to establish stakeholder perspectives towards the development of MI to include measures of pig health and welfare. In this paper the findings of semi-structured telephone interviews with 18 pig producers from the Republic of Ireland and Northern Ireland are presented.

**Results:**

Producers recognised the benefit of the utilisation of MI data as a health and welfare diagnostic tool. This acknowledgment, however, was undermined for some by dissatisfaction with the current system of MI information feedback, by trust and fairness concerns, and by concerns regarding the extent to which data would be used in the producers’ interests. Tolerance of certain animal welfare issues may also have a negative impact on how producers viewed the potential of MI data. The private veterinary practitioner was viewed as playing a vital role in assisting them with the interpretation of MI data for herd health planning.

**Conclusions:**

The development of positive relationships based on trust, commitment and satisfaction across the supply chain may help build a positive environment for the effective utilisation of MI data in improving pig health and welfare. The utilisation of MI as a diagnostic tool would benefit from the development of a communication strategy aimed at building positive relationships between stakeholders in the pig industry.

## Background

In the European Union (EU), meat inspection (MI) incorporates measures to conduct animal health surveillance, to protect public health and to ensure meat quality. As part of this process the detection of illness or injuries during the ante- and pathological lesions during the post-mortem period can lead to whole or partial condemnation of carcasses. There is increased interest in expanding the role of MI so that data collected could be used to better inform herd health and welfare management plans. In fact, examples already exist in the EU whereby the level of MI information provided, or the method by which this information is communicated, is specifically targeted at influencing management decisions in pig herds. For example, through a national animal health surveillance scheme launched in 1978, the Danish Pig Health Scheme identifies farms that exhibit high carcase condemnation rates and, in turn, offers the assistance of veterinary expertise [1, 2]. More recently, an industry-led initiative in Northern Ireland (NI) (Pig Grading Information System (PIGIS)) provides information on carcase grading and weight, and levels of total condemnation, to registered producers, while also allowing them to compare their results with top performing herds in these categories. Carcase Inspection Analysis (CIA) software is also under development in NI which will provide producers with real time and detailed access to meat inspectors’ assessments for their carcasses. Finally, in Great Britain, the Wholesome Pigs Scotland and BPEX Pig Health schemes record the presence of lesions from abattoir inspections, after which producers and their veterinarians are informed [3].

In order for MI data to play a useful role in on-farm management of both pig health and welfare, a number of factors need to be considered. Measures recorded at MI should be capable of identifying animal welfare outcomes such as tail lesions caused by tail biting. In addition, the information collected must be clearly communicated back to producers in a meaningful way, to enable producers to act on the information received. Ultimately, the success of the development of the MI process such that it can inform pig health and welfare management plans will hinge on stakeholder engagement with the process. In relation to pig welfare issues, previous research suggests that the level of importance producers attach to these issues will influence their willingness to adopt knowledge, information or technologies to address them [4].

Generally, gaps in knowledge and information explain why farmers often fail to implement, for example, recommended biosecurity control or welfare measures, or adopt new technologies [5, 6]. Such ‘information gaps’ are often explained by negative perceptions held by producers’ towards certain information sources [7]. Consequently, positive relations based on trust between the various sections of the supply chain are critical for the effective exchange and utilization of information [8–10]. In 2008, distrust and producer dissatisfaction linked to inadequate feedback between producers and processing plants, was identified as a problem for the development of the pig industry in the Republic of Ireland (ROI) [11]. In this case, distrust was related to the perceived inaccuracy of the carcase grading system, with some producers claiming inconsistencies in reporting between processor plants. Similar concerns are documented elsewhere regarding the reliability of the carcase grading processes and lack of price transparency (e.g. Germany; [12]). Research findings documenting inconsistencies in MI methods and recording practices both within and between jurisdictions [13, 14] does little to dissipate such trust issues. This suggests that a lack of trust of processors by pig producers may be a barrier to developing MI as a pig health and welfare diagnostic tool. Indeed, distrust between the public and communicators and regulators poses a fundamental challenge to effective communication and transfer of information and knowledge. Trust is characterised by numerous positive attributes, rather than just one defining feature [15]. For example, positive trust relations between communicators and the public are founded upon a perceived absence of bias; a commitment to due process; perceived objectivity, fairness and accuracy; competence and credibility; engagement and an expressed concern for public welfare [15, 16].

This study aims to establish perspectives of pig producers regarding the potential evolution of MI, data capture and utilisation to improve its contribution to diagnoses of pig health and welfare problems on farm. Part of this assessment also involved seeking producer views on pig health and welfare issues where feedback on MI data would be most useful. The paper forms part of a larger body of work which identified the perspectives of a range of stakeholders involved in pig production across the ROI and NI concerning the potential development of MI as an animal health and welfare diagnostic tool.

## Methods

### Study design

**Fig. 1:**
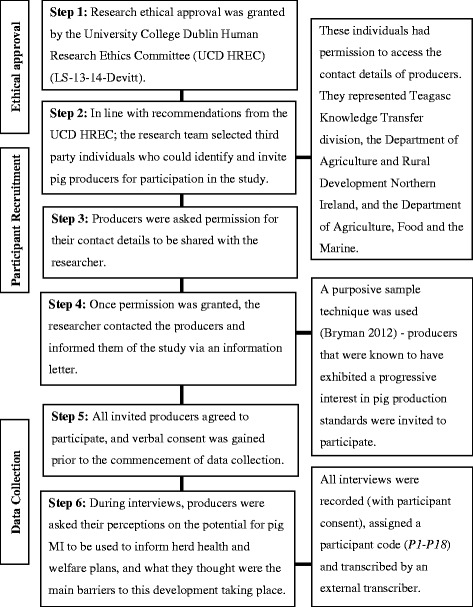
Flow diagram detailing methodological steps undertaken in the research. This figure details the six steps involved in the study methodology, and includes information on participant recruitment and data collection

This study used a qualitative research approach, comprising semi-structured interviews [17]. Figure 1 outlines the methodological steps taken by the research team. All members of the research team were involved in the development of research material, including interview protocols and information material administered to pig producers. The COREQ-32 (Consolidated criteria for reporting qualitative research) checklist was used to ensure quality control in the methodology, analysis and reporting of data.

### Participant recruitment and response

Eighteen telephone interviews were conducted with pig producers from various regions in the ROI and NI over three months in 2013. Four of these interviews were undertaken with producers who were taking part in the NI based Carcase Inspection Analysis (CIA) development program. Verbal consent to participate and consent to publish were obtained prior to interview.

### Data analysis

Identifiable information was removed from the transcripts prior to data analysis, after which transcripts were imported into a computer software analysis programme. Data were analysed thematically [18]. Thematic analysis is the most common form of analysis in qualitative research [18, 19]; and essentially, its aim is to develop thematic networks (based on the development of codes and categories) that analytically reflect the meaning of the data [18]. This approach is widely used in the health sciences; more recently it has been applied to the veterinary and agricultural sciences – for example, in Devitt and others [20, 21] in their qualitative research on farm animal welfare, in the work of Pritchard and others [22] on veterinarian awareness and understanding of biosecurity; and in a study involving farmers by Enticott and Vanclay’s [23] on animal health risks.

In this study, the data were analysed deductively and inductively. Deductively, the data was initially sorted according to the research questions, and these sections where then analysed inductively. Inductive analysis is particularly applicable to exploratory research as it is comprehensively data-driven, whereby the data itself generates new ideas and guides the process and direction of analysis [24]. Following well-established techniques presented by Attride-Stirling [18], a three-stage process was used. Following a period of familiarisation with the data, basic descriptive codes were applied to relevant segments of each transcript, generating a total of 84 codes comprising single words and brief descriptive summaries of what was being said in the data. These codes were then grouped analytically into second-level categories or organising themes that reflected a more conceptual explanation of the data, of which three were generated. Finally, organising themes were grouped into broad, conceptual overarching analytical or global themes, which forme conclusive interpretations of the text. With the support of illustrative quotes taken from the data set [25]; these global themes form the basis of the following results section. Agreement on the themes was reached between all authors.

## Results

The average farm size for the producer group was approximately 700 sows (ranging from 150 sows to 2,500 sows). The average herd size in Ireland is c. 600 sows so producers interviewed were representative of the average herd. Two key global themes were identified from the data analysis: i) producer aspirations for the use of MI data and ii) limitations that undermine the further development of MI data as an animal health and welfare diagnostic tool at the farm level. Table 1 summarises the saliency of these themes for producers.

**Table 1:** Prominence of themes across the producer data-set

Global theme	Organising theme	No. of data sources (*n* = 18) (no. of passages in each data source^a^)
Producer aspirations	Producer centred information	18 (32)
Limitations to the development of MI as a health and welfare diagnostic tool	Producer dissatisfaction and fairness concerns	13 (46)
	Perceptions of animal health and welfare	18 (39)

^a^This is the number of times the theme was mentioned in the data sources

### Aspirations; the need for producer-centred information

There was consensus amongst all producers (*n* = 18) that there is a potential benefit of assimilating MI data to inform herd health and welfare plans if it allowed them to gain a better understanding of the health and welfare issues on their farm. Ideally, for producers, feedback arising from MI should be provided for the purpose of producer interests, and must be practically applicable at the farm level:

‘*Once there’s a positive thing for the producers…. if* [meat inspector/processor] *is looking at my 100 pigs in the factory, and there was a problem of abscesses along the back, and if they came back to say that to me, and they had advice on tail biting as well, something proactive and positive to overcome the problem*…’. (P2)

Key areas of information mentioned as being of benefit for the producer included ‘*pleurisy*/*lung scores*/*pneumonia*’, ‘*poly-arthritis*’, and ‘*lesions*’, whether or not ‘*animal abuse*’ had occurred at some stage of production and transportation, ‘*injuries*’ and ‘*bruising*’. Additionally, some producers felt that greater explanation was required on reasons for carcase condemnation: ‘*a more transparent documentation on that specific load of pigs, there was one pig condemned, and half of another, and --- an element of honesty attached to it*’ (P16). Comments from four producers who were participants in the CIA development programme highlighted the value for them of receiving consistent information from the processor. These producers emphasised the significance of being able to review data and compare, in detail, the carcase-related status of their pigs over a period of time.

The private veterinary practitioner (PVP) featured strongly in the recalled experiences of *n* = 9 producers, when elaborating on contact with the processing plant, and in terms of their aspirations and expectations for the use of MI data as a health and welfare diagnostic tool. For example,

‘*I have an interest in finding out about the health of the pigs, so at the moment, I’d get my own vet to go into the factory and check the pigs. Because you are dealing with someone you know, someone who’s professional. You are dealing in the interest of keeping your business going well*’. (P11)

‘*If I want to know anything about that I have to get my own vet to go down and check the pigs out*’. (P2)

As a result of these producers close working relationship with their PVP, on further enquiry, they viewed them as having a positive role in helping them to decipher data for the individual farm context, and subsequently devise a herd health plan.

### Limitations; Producer dissatisfaction and fairness concerns

For the majority of ROI producers (*n* = 13), opinions on the potential benefit of receiving additional MI data were undermined by dissatisfaction with the current system of information provision from the processor. To illustrate, this producer commented on how the feedback he receives on reasons for condemnation, is of ‘*no use’*, explaining how ‘*we don’t know what pig it was, or which unit… other than you are minus €150 plus from your pay cheque*’ (P8)*.* Dissatisfaction with the stated reasons for condemnation received from the processor, featured strongly throughout the comments of *n* = 8 producers. To illustrate: ‘*Some vets have fetishes for certain reasons for condemning and they hone in on those…. There could be more standardisation in what we get back as producers, the differences between vets doesn’t make sense, and it makes you wonder how they carry out the meat inspection*’ (P4), with calls made for standardisation and more detailed information:

‘*The feedback really depends on the factory you go to. In* [first location]*, the main reason is pleurisy, but in the* [second location]*, it would be milk spot. I’d never get milk spot as a reason from the* [first location]… *I’ve no idea what’s going on… I don’t know why different response*’ (P5).

Producer dissatisfaction also extended to include concerns that MI data could be used unfairly against them (for *n* = 11), with a belief that producers would be financially penalised:

‘*What I don’t want to happen is a system that falls into place that will come back and bite me… as long as there’s no punishment attached to it*’ (P7).

‘*There’s huge interest in what’s going to happen to this information. There’s more tail biting in your place, the* [processor] *will dock you cent a kilo. We don’t get paid for positives. Everything is based on the negative*’ (P1).

‘*… I’m the one who’s losing out. My feeling on it* [use of MI data] *is that it would end up as a stick that could be brought back against us. And that’s why I’m asking, where is the feedback for me—where is the positive feedback for me? Especially when it’s coming from the processors, you know?*’ (P17).

Concerns over the perceived fairness of using MI data, reflect for this group of producers, a lack of concern for processor interests. To illustrate further, this producer explained that he would appreciate ‘*practical information, a phone call for example*’; however, he was of the opinion that ‘*the factory doesn’t care. They don’t want to be going slower to detect every issue… The factory will, if there’s information that can be used as a way of knocking down the price, that’s what they’d like*’ (P9). These beliefs and unfavourable perceptions influenced their reported willingness to pay for improved animal health and welfare related feedback arising from the capture and utilisation of MI data:

‘*If the detail of the inspections that there are in the plant at the moment are increased, I’d imagine that the producers are going to pay for it. It’s fair enough to get the feedback—I’d have a bit of a trust issue there, as to getting that feedback and in terms of how reflective it is… there’s a lot of work needed to be done before we’d consider paying, we need to see the benefit*’(P11).

### Limitations; Producer perceptions of animal health and welfare issues

While information from the abattoir on the extent of abscesses, tail biting, lameness and other health and welfare related issues was deemed useful, levels of interest attached to the prioritisation of different types of issues differed across the producer group. These differences were related to producers’ perceptions of specific animal welfare and health issues and the importance they attached to welfare issues at the farm level. Producers were specifically asked about tail biting and there were varying opinions on its priority as an issue for concern. To illustrate, this producer reported that he knew the causes of tail biting and there was little the abattoir could inform on. Yet, he went on to outline the need for ‘*a good comprehensive report on each load of pigs – if the pigs had any condemnation in it, and what was exactly wrong*’ (P8) ; a comment which suggests a failure to link issues such as tail biting and eventual pig meat condemnation. Other producers reported a need to know the causes:

‘*I’d love to know what causes it, because it’s not good to see an animal suffer. When an animal suffers it’s not making me any money either, and it’s no good to me*’ (P5)

‘*It would be interesting to have results on tail biting, we could correlate it with the time of year. You don’t record it because its irregular but it would be good to get a better understanding on it, to get a better idea of the problem*’ (P17).

For some (*n* = 5), issues such as tail biting and its relevance to the development of MI as a welfare and health diagnostic tool were explicitly reported as being less of a priority. This was because of what was described as its irregular nature on individual farms, the fact that it fell within a threshold that producers felt they could do nothing about, as deemed to be caused by environmental factors that were outside of the control of the producer:

‘*You’d get an odd animal with an abscess from tail biting—you don’t think too much about it, as long as it doesn’t happen too much*’ (P13)

‘*At certain times of the year you might have a bit of a flare-up, the tail biting is just an irritation. If the pigs are in any way irritated. This warm weather… It might upset the pigs, and you might get a flare-up of tail biting… on its own, since we started cutting the tails shorter, has stopped that problem*’. (P9).

The relationship between perceptions of animal health and welfare and the perceived relevancy of MI data was also apparent in the following comments:

‘*I am on the farm myself, I load pigs myself, and so I have an idea of the standard of the pigs. You will always have a bit of lameness, if you are producing 500 pigs a week; you will always three or four lame pigs a week*’. (P12)

‘*I know what’s causing the issues I have. But I can’t do anything about them. I’m getting septic pleurisy. Those are pigs that have prolapsed. The bad weather. There’s nothing to be done about that… and I got it down to a level that I can live with like, but it’s still there*’. (P9)

There was evidence to suggest minor distinctions in perceptions of animal health and welfare between producers (in both jurisdictions), and between those participating in the CIA programme. It was reported that being able to assess the frequency of certain health issues allowed CIA producers to correlate with contributing factors on the farm, thus allowing them to rationalise the causes and understand the extent of the issue on their farm.

## Discussion

### Overview

Previously Harley and others [13] suggested that ante- and post-mortem MI results could be developed into a health and welfare diagnostic tool and used by producers to inform herd health and welfare plans. Understanding pig producer perspectives in the pig sector is a key component to developing new systems of data capture and utilisation in pig meat production. In order to achieve this objective, telephone interviews were conducted with pig producers as part of a larger social science study that also involved interviews and focus groups with a range of stakeholders involved in the pig production industry across ROI and NI.

This study identified two global themes attributable to the attitudes of pig producers towards the development of MI as a health and welfare diagnostic tool. The first theme related to producer aspirations, with general agreement among all producers on the potential usefulness and benefit of such a tool. Highlighting some of the benefits of consistent feedback to producers, the advantages of participation in the CIA development programme in NI allowed for a greater producer understanding of the frequency and related seriousness of particular health-related issues. However, dissatisfaction for a number of producers, with the current system of information provision from the processor, and related distrust over the reasons currently provided for carcase condemnation undermine the potential usefulness of MI as a health and welfare diagnostic tool, particularly among those not already participating in programmes such as the CIA. Producer dissatisfaction also extended to include concerns that MI data could be used by the regulatory authority as a mechanism to impose penalties if their pigs had high levels of welfare lesions. This reflects fears that MI data would be used as a surveillance, rather than diagnostic, tool. The former infers monitoring while a ‘diagnostic tool’ is one which can be used to identify trends in patterns and prevalence of health and welfare lesions. Clearly the latter could be a useful management tool for producers and/or their PVP to support continuous improvement in pig welfare and reduce financial losses at slaughter due to carcass condemnation. The lack of importance, and a poor understanding of certain welfare issues such as tail biting among some producers was a third important constraint to the development of MI as a pig welfare and health diagnostic tool. The remainder of this section of the paper discusses the implications for realising the value of MI data as an animal health and welfare diagnostic tool.

### Challenges in realising the value of MI data at farm level

A number of the potential challenges to realising the value of MI data at the farm level outlined in the results are worthy of further attention, in particular producer dissatisfaction, trust issues and fairness concerns. These issues are noted elsewhere [11, 13, 14]. Although they present challenges to realising the value of MI data at farm level, these challenges are not unique to the particular topic under review. Several authors identified mistrust between various stakeholder groups as presenting a challenging obstacle for the improvement of bio-security at farm level [26], promoting producer participation in welfare schemes [4], and in realising the effectiveness of agri-food chains [27]. Clearly, trust between the various sections of the supply chain is essential for the effective exchange and utilization of information [9], presumed credibility of information content, and subsequent willingness to cooperate and comply with information recommendations [28].

If a source of distrust exists, it will prove difficult to form and improve more favourable relations between relevant stakeholders [27]. This relational component can be influenced by the extent to which power is distributed along the production chain as well as by the prevailing economic situation [29]. For example, the economic conditions at a particular point in time may see various participants along the production chain competing with each other for a diminishing profit margin; thus leading to concerns over fairness between producers and processors [12, 27]. Notably, the study in hand was conducted at a time of economic difficulty in Ireland, the conditions of which may have influenced concerns over perceived fairness, as noted by producers, the reported willingness to pay for receipt of MI data, and whether or not such would yield any financial benefits for the producer. Indeed, concerns over fairness cannot be detached from the wider trust framework – this is because, as earlier reported; in general, trust between the public and communicators/regulators is founded upon (in addition to other features), perceived fairness and objectivity [15, 16].

In this study, welfare problems such as tail biting are presented as acceptable when within a tolerable, manageable level; though some producers express a desire to determine the causes. The opinions of some producers regarding the causes of tail biting were somewhat misguided. Similar to Bracke [30], producers believed the weather was a dominant cause of tail biting, despite scientific studies showing otherwise [31]. Indeed producers perceived locus of control (that is, the extent to which they believe they are in control of an event of incident occurring) helps explain their attitude towards certain health and welfare problems, such as tail biting. Hence, as producers believe that the weather is an important risk factor for tail biting and they cannot control the weather, they believe that they cannot control tail biting. Similarly, Kauppinen [32] found that the degree to which farmers believed they had behavioural control over animal welfare improvement practices was linked with farm productivity. Though writing on disease risk management, similar results are reported by Garforth [33]. Decisions to implement a specific control measure are influenced by farmer attitudes to risk, the practicality of implementing the control measure, and the credibility they ascribe to information and advice received [33]. This latter feature is important to this study because it supports earlier points made, by showing the necessity of a favourable perception of the various roles involved in pig meat production and information dissemination, in order for the information received to be seen as credible, legitimate, and worthy of acting on.

Clearly, producer willingness to engage in animal welfare related schemes is influenced by how they define and attach importance to animal welfare issues [4], their beliefs reported in our study around certain animal welfare issues may limit their perceived usefulness of MI data in informing herd health and welfare plans. In the absence of regular data highlighting the frequency of tail-biting and docking related injuries, it may be difficult for pig producers to develop a fuller understanding of its impacts. For example, Harley [34] demonstrated the financial losses for pig producers (and processors) recorded at slaughter, arising from tail-biting. Market or processing-led incentives could be used to incentivise producers to deliver pigs with intact tails [30]. However, trusted, credible and consistent information is also crucial in positively informing producer attitudes and behaviour. Drawing on the risk communication literature; if levels of trust are high, the public are more willing to refer on expert judgement when making certain decisions [35, 36]; with higher degrees of trust across the industry, producers may be more receptive to receiving advice on certain health and welfare issues.

### Limitations of the study

Careful consideration was applied to the recruitment of producers as based on the requirements established during the ethical consent process (Fig. 1), however this resulted in an inbuilt bias as recruiters selected producers that they believed had an active interest in pig production matters and were likely to consent to participation. The authors acknowledge the small sample size; while on one level, the views expressed cannot be representative of the producer population as a whole, a high degree of consensus and reiteration of perceptions was expressed within the group, especially among those in ROI. Indeed, one can assume that as the producers involved in this study were keen to express and discuss their opinions, the results presented can therefore be said to reflect the opinions of producers most motivated to engage and improve animal health and welfare on their farms. Furthermore, the herd sizes of the producers interviewed in this study mirrored the average herd size for producers in ROI and NI. However, the following recommendations for realising the potential of MI data at farm level may prove less effective for less interested producers – for example, less interested producers may be less willing to partner with their PVP in herd planning. Different perspectives on trust may also exist.

### Recommendations for realising the potential of MI data in herd health planning

The development of positive relationships based on trust, commitment and satisfaction between the supplier and processor may help provide a more favourable environment in which MI data can be received positively at the farm level. This can, in part, be achieved by developing personal bonds based on active engagement and partnership between all stakeholders, while building the wider level of credibility in which information is perceived by producers [9, 29]. Furthermore, given the centrality of presumed objectivity, accuracy and consistency in forming trust relations; improved standardisation of terminology used in the feedback and information provided to producers, and better training of meat inspection roles, may be required. This need for consistency is made clear in the comments of those participating in the CIA programme - these producers emphasised the value of consistent information and the ability to review and chart progress over time.

Realising the full potential of MI data in herd health and welfare planning at the farm level may also require emphasising the centrality of the PVP in communicating information and working with producers. Using MI data as a diagnostic tool to identify trends in welfare lesions could be a useful management tool for producers and/or their PVP to support continuous improvement in pig welfare and reduce financial losses at slaughter due to carcass condemnation. Across a number of agricultural sectors, positive relationships between farmers and their veterinarian form a key component in information dissemination and capacity building [6, 10, 33]. In this study, producers reported that the PVP had a central role on their farms. With respect to the potential use of MI data, the PVP was also identified as being potentially important in working with producers to address health and welfare issues identified through MI data. This finding mirrors research elsewhere. For example, veterinarians are often relied on exclusively for communicating disease-related information at farm level [7] and are seen as a trusted source of information for helping producers improve on-farm management [10, 26].

Considering the challenges identified in this paper, the further development and utilisation of MI data as a health and welfare diagnostic tool may require the implementation of an effective communication strategy. Such an approach could have at its core, the objective of building trust and a culture of partnership between all stakeholders, while informing producers on the implications of certain pig-related health and welfare problems, and enabling and empowering them to see the producer-centred benefits of MI data. In line with general recommendations put forward by Garforth [33], the information communicated may need to be targeted, and involve some component of PVP participation. Indeed, this study looks only at perceptions around the usefulness of information but does not consider factors which may impact on the actual behavioural implementation of health and welfare measures informed by MI data. In order to achieve full effectiveness, any communication around the use of MI data in informing herd health plans should also consider the factors that exist at farm-level, such as producer attitudes and perceptions around risk, behavioural motivations, sense of self-efficacy, and competence in carrying out animal health and welfare actions [33, 37–39].

## Conclusions

In conclusion, the development of MI as an animal health and welfare diagnotic tool can bring benefits to pig producers, for herd health planning. However, in order for this potential to be fully realised, important ingredients such as consistency in approach, trust, and satisfaction across the supply chain based on positive relationships between all actors, is required.
